# Association between non‐melanoma skin cancer and risk of fractures

**DOI:** 10.1002/ski2.309

**Published:** 2023-11-02

**Authors:** Vaishali Mittal, Eric M. Anderson, Gina P. Kwon, Marcia L. Stefanick, Katherine J. Ransohoff, Haley K. Hedlin, Jean Wactascki‐Wende, Ashley S. Wysong, Jean Y. Tang

**Affiliations:** ^1^ Stanford Hospital and Clinics Redwood City California USA; ^2^ Stanford Prevention Research Centre Stanford University School of Medicine Stanford California USA; ^3^ Quantitative Science Unit Department of Medicine Stanford University Stanford California USA; ^4^ School of Public Health University of Buffalo Buffalo New York USA; ^5^ Department of Dermatology University of Nebraska Medical Center Omaha Nebraska USA

Dear Editor,

In recent decades, there has been a dramatic increase in the incidence of both malignant melanoma and non‐melanoma skin cancer (NMSC), especially among women.^1^ Ultraviolet light light is the major risk factor for the development of NMSC, and sun protection is recommended to lower NSMC risk, especially in patients with a prior history of skin cancer. However, there is debate amongst health care providers about whether sun protection measures (seeking shade, sunscreen use, wearing sunprotective clothing/hat) to prevent skin cancer should be moderated. It is theorized that decreased sun exposure leads to decreased skin synthesis of vitamin D and therefore, increased fracture risk.^2^ We aim to investigate the relationship between NMSC and future bone fractures among the Women's Health Initiative (WHI) population, a large prospective cohort of postmenopausal women.^3^

Our data is derived from the observational study component of the WHI, an 11‐year study of women ages 50–79 who answered surveys about sun protective habits, oral vitamin D intake, physical activity, osteoporosis, and other risk factors for fractures. Our cohort (*n* = 71 759) includes data from over 40 clinical centres across the U.S. and years 1992–2007.^3^ We restricted our cohort to white participants because skin cancers are rare among the non‐white population,^4^ those who completed a sun exposure survey at year four, and excluded patients with a history of hip replacement at baseline. Women with or without a baseline history of NMSC were analysed for the development of lower arm, hip, and spine fractures. New diagnosis of spine and arm fractures was self‐reported, and hip fractures were confirmed by medical records. Baseline characteristics of women who did and did not develop fractures were compared by Chi‐square tests or analysis of variance for continuous variables. Adjusted hazard ratios (HRs) and 95% confidence intervals (CIs) were computed from Cox proportional hazards analyses.

Our results show that there were 4289 women with a baseline history of NMSC. During a mean follow‐up of 11 years, there were 7765 incident fractures among all patients with NMSC. Women with a history of NMSC were more likely to be over the age of 70 (33.5% vs. 23.0%, *p* < 0.0001), meet or exceed recommended daily calcium intake (50.2% vs. 44.5%, *p* < 0.0001), and have a daily vitamin D intake greater than 500 IU (25.4% vs. 21.1, *p* < 0.0001). Patients with a history of NMSC had more than 2 h of daily summer sun exposure as a child (72.0% vs. 69.8%, *p* < 0.0001) and reported 475–500 Langleys of sun exposure (25.7% vs. 22.4%, *p* < 0.0001). Patients with a history of NMSC practiced more sun protection, as measured by more than 2 total hours of daily current summer sun exposure (16.2% vs. 18.9%, *p* < 0.0001) and increased sunscreen use (64.3% vs. 50.0%, *p* < 0.0001). They were also more likely to have a history of osteoporosis (12.3% vs. 8.5%, *p* < 0.0001) and prior fracture (45.7% vs. 38.8%, *p* < 0.0001). Table 1 shows the results of Cox proportional hazards models for hip, spine, and lower arm fractures in women with and without a history of NMSC. In adjusted models, a history of NMSC was only associated with increased risk of only lower arm fractures (HR 1.19, 1.02–1.38 95% CI, *p* = 0.02). Importantly, women with history of NMSC had no increased risk of hip or spine fracture.

**TABLE 1:** Incidence rate of hip, spine, and lower arm fractures in the WHI Observational Study (*N* = 71 759) in post‐menopausal women with and without a history of NMSC.

Outcome	Number of incident fractures (%)		Adjusted hazard ratio (95% CI)	*p*‐value
	No history of NMSC (*N* = 67 470)	History of NMSC (*N* = 4289)		
Hip fracture	1565 (2.32)	150 (3.50)	1.14 (0.92–1.42)	*0.24*
Spine fracture	2157 (3.20)	166 (3.87)	0.98 (0.81–1.19)	*0.85*
Lower arm fracture	3466 (5.14)	261 (6.09)	1.19 (1.02–1.38)	*0.02*

*Note*: Adjusted Cox proportional hazards models were performed for fracture by site. Potential differential effects across subgroups of key risk factors for skin cancer were tested individually with the use of a likelihood ratio test for interaction between the risk factor and treatment assignment after including both as main effects. Adjusted models included BMI, smoking status, history of fracture, physical activity in Metabolic Equivalent Task (MET), history of fracture over age 55, falls in the past year, arthritis, osteoporosis, bisphosphonate drug use, hormone use, vitamin D intake, and calcium intake, all at baseline, and age, education, Langley's of exposure of clinical centre, age at menopause, maternal history of hip fracture, and greater than moderate alcohol consumption. Italic *p* values 〈 0.05 are considered statistically significant.

Abbreviations: NMSC, non‐melanoma skin cancer; WHI, Women's Health Initiative.

We previously found that elderly men with a history of NMSC practice photoprotection measures, have lower vitamin D levels, and therefore are potentially predisposed to increased fractures.^5^ However, our analysis of postmenopausal women found that levels of serum 25(OH)D are similar in those with or without history of NMSC.^6^ Recent literature has not confirmed the association between sun protection and vitamin D deficiency, and a large randomized, controlled trial showed that vitamin D supplementation does not significantly lower risk of fractures in older adults.^7^, ^8^ In line with this new evidence, we found that a history of NMSC is not associated with significantly higher fracture risk, especially hip and spine fractures. Although we did find that women with NMSC have a 19% higher risk of only lower arm fractures, we hypothesise that this observation might not be directly linked to photoprotective measures. Instead, it could be attributed to an elevated susceptibility to falls since lower arm fractures are often common with such incidents.^9^ Although we adjusted for occurrences of falls within the past year, we did not factor in a measure of fall risk. Our adjustment was limited to considering recent falls as a potential confounder, rather than encompassing the wider array of factors that collectively contribute to an individual's overall susceptibility to falling. Notably, the WHI cohort comprises elderly women, a demographic potentially more prone to falls, consequently leading to a higher occurrence of lower arm fractures. Further investigation within the WHI cohort is necessary to understand the factors that contribute to fracture rates to fully understand the association between a history of NMSC and the occurrence of lower arm fractures. In conclusion, our findings are consistent with newer evidence^7^, ^8^ that suggest dermatologists do not need to moderate sun protective measures in patients with NMSC to mitigate against fracture risk. Our study demonstrates that despite the NMSC cohort adhering to sun protection practices, they did not exhibit an elevated risk of hip or spine fractures.

## CONFLICT OF INTEREST STATEMENT

The authors declare no conflicts of interest.

## FUNDING INFORMATION

This study was supported by the National Heart Lung and Blood Institute.

## AUTHOR CONTRIBUTIONS

**Vaishali Mittal**: Visualization (lead); writing—review and editing (lead). **Eric M. Anderson**: Formal analysis (equal); methodology (equal); writing—original draft (lead). **Gina P. Kwon**: Formal analysis (equal); writing—original draft (equal); writing—review and editing (equal). **Marcia L. Stefanik**: Conceptualization (equal); formal analysis (equal); writing—review and editing (supporting). **Katherine J. Ransohoff**: Writing—review and editing (equal). **Haley K. Hedlin**: Formal analysis (equal). **Jean Wactascki‐Wende**: Writing—review and editing (supporting). **Ashley S. Wysong**: Conceptualization (equal); writing—review and editing (equal). **Jean Y. Tang**: Conceptualization (lead); methodology (equal); supervision (lead); visualization (equal).

## ETHICS STATEMENT

Not applicable.

## Data Availability

The data that support the findings of this study are available from the Women's Health Initiative. The WHI dataset is available with the permission of the WHI to researchers with approved research proposals. More information about access to the WHI datasets can be found at https://sp.whi.org/researchers/data/Pages/Home.aspx.
